# Connecting the Dots: Resolving the Bone Marrow Niche Heterogeneity

**DOI:** 10.3389/fcell.2021.622519

**Published:** 2021-03-12

**Authors:** Igor Dolgalev, Anastasia N. Tikhonova

**Affiliations:** ^1^Applied Bioinformatics Laboratories, NYU School of Medicine, New York, NY, United States; ^2^Princess Margaret Cancer Centre, University of Toronto, Toronto, ON, Canada

**Keywords:** microenvironment, stromal – hematopoietic cells interactions, bone marrow, cell-to cell communication, hematopoiesis, single-cell RNA-seq (scRNA-seq), single-cell ‘omics

## Abstract

Single-cell sequencing approaches have transformed our understanding of stem cell systems, including hematopoiesis and its niche within the bone marrow. Recent reports examined the bone marrow microenvironment at single-cell resolution at steady state, following chemotherapy treatment, leukemic onset, and aging. These rapid advancements significantly informed our understanding of bone marrow niche heterogeneity. However, inconsistent representation and nomenclature among the studies hinder a comprehensive interpretation of this body of work. Here, we review recent reports interrogating bone marrow niche architecture and present an integrated overview of the published datasets.

## Hematopoiesis

Hematopoiesis is a continuous process of generating blood and immune cells and is one of the best-studied stem cell systems in modern biology (Weissman, 2000). Since the discovery of hematopoietic stem cells (HSCs), the field has been driven by technological advances such as flow cytometry, mass cytometry, high-resolution microscopy, and now single-cell sequencing approaches. *In vitro* approaches, such as colony-forming assays, defined the intermediate stages between a rare population of multipotent hematopoietic stem cells and the terminally differentiated cell populations. *In vivo*, early hematopoietic progenitors were further classified as long-term HSCs (LT-HSCs) capable of self-renewal and unlimited differentiation potential and multipotent progenitors (MPPs), characterized by limited self-renewal capacity (Reya et al., 2001). The MPPs have been further separated into myeloid-biased MPP2 and MPP3 as well as lymphoid-biased MPP4 subsets that differentiate to lineage-restricted common myeloid progenitors (CMPs), granulocyte/macrophage progenitors (GMPs), and common lymphoid progenitors (CLPs) (Pietras et al., 2015). Extensive literature continued to define the complexity of hematopoietic differentiation, incorporating further subpopulations and subdivision of downstream progenitors (Cabezas-Wallscheid et al., 2014; Liggett and Sankaran, 2020).

The last decade has witnessed the introduction and wide-scale adoption of high-throughput approaches, including single-cell RNA sequencing (scRNA-seq). Unlike more traditional flow or mass cytometry methods that are limited by predefined markers, scRNA-seq technologies simultaneously capture thousands of genes in each cell, providing an unbiased characterization of their transcriptional diversity even within phenotypically homogenous populations. Notably, a major advantage of single-cell transcriptional profiling is the identification of novel populations and states as well as shifts in their relevant frequencies (Kowalczyk et al., 2015).

## Hematopoietic Differentiation at Single-Cell Resolution

In one of the earlier large-scale single-cell studies, Paul et al. (2015) characterized 2,730 myeloid progenitor cells. Clustering analysis based on an expectation maximization (EM)-like procedure resulted in 19 subpopulations, including groups corresponding to known erythrocyte, monocyte, and neutrophil progenitors, as well as more transcriptionally heterogeneous clusters. The data showed that the traditional GMP and CMP definitions include several subpopulations with distinct lineage commitment transcriptional profiles.

Nestorowa et al. (2016) comprehensively profiled individually sorted 1,656 murine hematopoietic stem and progenitor cells (HSPCs), with an average of 6,558 protein-coding genes detected per cell. The study utilized the diffusion maps dimensionality reduction technique (Haghverdi et al., 2015; Angerer et al., 2016) to visualize and interpret the continuous processes of early hematopoietic differentiation. The cells were assigned to 12 commonly sorted HSPC phenotypes based on surface markers. When displayed on the diffusion map, all populations, except CMPs, localized within defined regions. The relative ordering of populations within the diffusion map was in agreement with the established hematopoietic hierarchy and further showed intermediate transition zones containing transcriptionally similar cells that traditional gating assigned to differing phenotypes. Interestingly, the single-cell analysis revealed that the three broad trajectories defined by the diffusion map begin to diverge immediately following the LT-HSC stage.

The findings were extended to the human system. Velten et al. (2017) combined transcriptomic and functional single-cell data to investigate the dynamics of lineage commitment of individual cells. Healthy human HSPCs were defined by the absence of lineage markers and CD34 expression (Lin^–^CD34^+^). 1,413 HSPCs from the bone marrow of two individuals were profiled by scRNA-seq and 2,038 cells individually cultured *ex vivo* were used to determine lineage potential. The transcriptomic and functional datasets were integrated based on surface marker expression. Clustering within the Lin^–^CD34^+^CD38^–^ compartment that includes HSCs and their immediate progeny, such as MPPs, was unstable and the cells formed a continuously connected unit. In contrast, more differentiated Lin^–^CD34^+^CD38^+^ progenitors formed clusters corresponding to defined progenitor populations. The scRNA-seq data separated the cells into a continuum of low-primed undifferentiated (CLOUD)-HSPCs and discrete populations of restricted progenitors associated with increased CD38 expression. Analysis of the discrete Lin^–^CD34^+^CD38^+^ sub-populations identified the major branches of hematopoiesis, including B-cell progenitors, megakaryocyte/erythrocyte committed progenitors, neutrophil-primed progenitors, monocyte/dendritic cell progenitors, and eosinophil/basophil/mast cell progenitors. The authors developed a dimensionality reduction technique STEMNET to view the transformation from HSCs to the lineage-restricted progenitors by placing each cell along a trajectory from the least-primed HSCs in the center toward one of the six restricted progenitor populations in the corners of a simplex. The authors proposed a model in which distinct lineages emerge from CLOUD-HSPCs rather than a series of discrete progenitors. Zheng et al. (2018) profiled 21,306 CD34^+^ progenitor cells from human cord blood across five biological replicates without enrichment or depletion for specific lineages. The analysis revealed previously defined progenitor populations in addition to the intermediate continuous states. The authors additionally integrated the data with the previous bone marrow dataset from Velten et al. to show the high concordance between the two systems.

Pellin et al. (2019) transcriptionally mapped the fates of the early human hematopoietic progenitors using 6,011 CD34^+^ and 15,401 Lin^–^ single cells profiled with the inDrops protocol. The hierarchical continuum of states that branch out toward cells expressing established lineage signatures was visualized using SPRING (Weinreb et al., 2018a). This two-dimensional force-directed graph layout generates a graph of nodes representing cells connected to their nearest neighbors in high-dimensional gene expression space. The organization of the data broadly segregated the cells into known immunophenotypic subpopulations and detected extensive transcriptional heterogeneity among HSPCs. The analysis suggested a structured hierarchy of fate decisions rather than a single step transition from undifferentiated HSPCs to committed states. This organizational structure was confirmed by inferred transcriptional trajectories as well as by population balance analysis (PBA) (Tusi et al., 2018), which in addition to providing a static continuum description of cell states aims to infer dynamic properties such as fate potential. This work confirmed a continuous hierarchical organizational structure from HSPCs to lineage-committed states not fully defined by traditional immunophenotyping. Importantly, the authors compared the human and mouse HSPCs, revealing a comparable branching structure within the two species despite differences in cell surface markers commonly used to isolate the various subpopulations.

Collectively these studies were able to isolate and define highly granular HSPC populations. They highlighted the transitional phases of hematopoietic differentiation, building on the traditional tree-like model of hematopoiesis toward a more fluid path of stem cell differentiation (Laurenti and Göttgens, 2018; Watcham et al., 2019).

## Bone Marrow Niche

Postnatally, HSCs reside in the bone marrow. While substantial progress has been made in understanding the hematopoietic hierarchy, the extrinsic factors guiding HSC self-renewal and differentiation are less defined. The bone marrow is a complex organ composed of numerous cell types that regulate HSC function through physical and biochemical interactions (Pinho and Frenette, 2019). The discovery that SLAM family surface receptors CD150, CD244, and CD48 were differentially expressed among HSPC populations in a way that can track developmental potential made it possible to image and localize those populations within the bone marrow (Kiel et al., 2005). Many HSCs were found associated with sinusoidal endothelial cells (ECs) and the endosteum (Kiel et al., 2005) as well as in contact with Cxcl12-abundant reticular (CAR) cells surrounding ECs (Sugiyama et al., 2006). An extensive network of sinusoids in the central marrow allows the hematopoietic cells to transport in and out of the bone marrow. In addition to serving as a barrier, EC-specific deletion of Scf (Kitl), Cxcl12, Jag1, and Dll4 impacted HSC quiescence, differentiation, and localization (Ding et al., 2012; Ding and Morrison, 2013; Poulos et al., 2013). Stromal cells make up another major constituent and multiple sub-populations have been previously described, including Lepr^+^ MSPCs, NG2^+^ (CSPG4, a pericyte marker) cells, and CAR cells. NG2^+^ cells unsheathe arterioles have been previously proposed to maintain HSC quiescence (Kunisaki et al., 2013). Osteoblasts derived from the bone marrow MSPCs were previously shown to support early lymphoid progenitors (Ding and Morrison, 2013).

Recent studies extended single-cell transcriptomic profiling to the non-hematopoietic compartment of the murine bone marrow to further understand the combination of signals and their cellular source responsible for HSC maintenance and differentiation. As the frequencies of bone marrow cell types vary by orders of magnitude (Gomariz et al., 2018), some groups utilized an approach of depleting abundant hematopoietic cell types to isolate the niche populations in an unbiased manner while others devised a positive selection approach to focus on previously identified subsets (Table 1).

**TABLE 1:** Recent scRNA-seq studies profiling the non-hematopoietic compartment of the murine bone marrow.

Publication	Isolation approach	Source	Digestion	Enzymes	Library platform	Analyzed cells
Baccin et al., 2020	- Negative selection approach. - Bone marrow and bone fractions.	Femurs, tibiae, hips and spines	Undigested and enzymatically digested	Collagenase II and Dispase	10× Genomics, FACS-indexed, LCM-seq	7,497 (2,540 non-hematopoietic)
Baryawno et al., 2019	- Negative selection approach. - Ter119^LOW^,CD71^LOW^, CD45^–^, CD3^–^, B220^–^, CD19^–^, Gr-1^–^, Cd11b^–^. - Bone marrow and bone fractions.	Femurs and tibia	Enzymatic digestion	STEMxyme1 and Dispase II	10× Genomics	20,896
Tikhonova et al., 2019	- Positive selection approach. - VEcad-tdT, Lepr-tdT, and Col2.3-tdT animals. - Sorted tdT^+^CD45^LOW^TER119^LOW^. - Lepr-tdT/VEcad-tdT—Bone marrow and Col2.3-tdT—Bone marrow and bone fractions.	Femurs, tibias, and ileums	Enzymatic digestion	Liberase and DNaseI	10× Genomics	9,622
Wolock et al., 2019	- Negative selection approach. - CD45^LOW^, Ter119^LOW^, CD31^–^ - Bone marrow and bone fractions.	Femurs, tibiae, and pelvic bones	Enzymatic digestion	Collagenase/Dispase	inDrops	2,847
Zhong et al., 2020	- Positive Selection approach. - Col2-tdT Bone marrow.	Long bones	Enzymatic digestion	Not indicated	10× Genomics	13,759

Our group took advantage of lineage-specific Cre-labeling to profile 9,622 cells representing major niche subsets previously shown to play a role in hematopoiesis using the 10× Genomics platform (Tikhonova et al., 2019). Clustering analysis detected two endothelial, four perivascular, and three osteo-lineage subpopulations, validated using orthogonal approaches such as immunofluorescence or flow cytometry-based on the identified biomarkers. The vascular endothelial subset marked by expression of *Cdh5* (VE-Cadherin) contained cells representing *Stab2*^+^ and *Flt4*^+^ sinusoidal capillaries and a smaller (12% of ECs) fraction of *Ly6a*^+^ and *Cd34*^+^ arterial cells. The *Lepr*^+^ perivascular compartment consisted primarily of adipo-primed mesenchymal cells characterized by high expression of *Cxcl12* and adipogenic markers such as *Adipoq* and *Lpl* as well as osteo-primed cells defined by gradually increasing expression of osteogenic genes such as *Bglap* and *Spp1*. This was a surprising finding, given that the bone of young animals contains few adipocytes. The osteo-lineage subset was comprised of three transcriptionally distinct clusters representing osteoblasts, chondrocytes, and fibroblasts. To infer the relationship between the mesenchymal lineage cells, the developmental trajectory was reconstructed using Monocle (Trapnell et al., 2014). Pseudotime ordering of the perivascular and terminally differentiated osteo-lineage cells revealed a continuum of cellular states with progressively increasing levels of expression of adipogenic markers toward one endpoint and osteogenic markers toward the opposite end. The adipo-primed sub-populations were enriched for previously identified human bone marrow mesenchymal stem cell gene signature (Ghazanfari et al., 2016), and functional studies showed higher activity of fibroblastic colony-forming units. In contrast to osteo-rimed progenitors that were tdTomato^+^ but expressed lower levels of *Lepr*, adipo-primed progenitors were both high in *Lepr* expression and tdTomato^+^. Taken together these findings indicate that early mesenchymal progenitors reside within adipo-gene expressing *Lepr*^+^ MSPC fraction.

Wolock et al. (2019) examined the mesenchymal lineage cells to determine the relations and hierarchies of stromal progenitors as they differentiate toward the mature bone, fat, and cartilage cells. 2,847 sorted non-hematopoietic (CD45^–^Ter119^–^) and non-endothelial (CD31^–^) cells were profiled by 3′ droplet-based inDrop scRNA-seq with a median of 736 detected genes per a cell. Spectral clustering identified 7 clusters labeled as MSC, adipocyte progenitors, pre-adipocytes, osteoblast/chondrocyte progenitors, pre-osteoblast/chondrocytes, pro-osteoblasts, and pro-chondrocytes. SPRING visualization exposed a transcriptional continuum across the adipogenic and osteogenic branches. Differentiation trajectories were inferred with Velocyto (La Manno et al., 2018), a method to quantify the relationship between the abundance of precursor and mature mRNA, predicting individual cells’ future state. RNA velocity analysis suggested that the MSC subpopulation was atop the differentiation hierarchy with branches toward the pre-adipocyte, pro-osteoblast, and pro-chondrocyte endpoints. PBA was utilized as an independent methodology to resolve each of the three lineages’ average transcriptional trajectory and order genes based on their expression pattern along those trajectories.

Utilizing a negative selection approach, Baryawno et al. (2019) profiled 20,896 bone marrow Lin- cells and identified 17 clusters originating from bone and bone marrow, including endothelial cells, MSCs, osteolineage cells, chondrocytes, fibroblasts, and pericytes. Multiple methods were utilized to resolve differentiation relationships between the populations, including the correlation of expression profiles between clusters and diffusion map analyses. The clusters’ connectivity was evaluated with partition-based graph abstraction (PAGA) that generates a graph-like map of data maintaining both its continuous and disconnected structure. Three endothelial clusters were identified, spanning a transcriptional continuum from Flt4 + sinusoidal to Ly6a + arteriolar sub-populations. The latter additionally had a sub-population of cells from endosteal and bone marrow arteries, marked by expression of *Vwf* and *Kitl*. Mesenchymal cells, classified based on the expression of *Lepr* and *Cxcl12*, were further divided into four subsets with varying levels of expression of those markers. One was characterized by osteo-lineage markers *Sp7* and *Alpl*, indicating differentiation toward that lineage. This relationship was further profiled by analyzing the two osteolineage clusters, one covering a range of differentiation states and a transcriptionally distinct subset of mostly cells from the bone fraction. An additional distinct subpopulation of perivascular mesenchymal stromal cells and pericytes was identified with low levels of Lepr but high levels of *Nes* and NG2 (*Cspg4*) as well as pericytes markers *Acta2*, *Myh11*, and *Mcam*.

Baccin et al. (2020) combined single-cell with spatially resolved transcriptomics. To compensate for the highly variable abundance of cell types within the bone marrow, undigested bone marrow or enzymatically digested bones were depleted or enriched for populations of interest. The final dataset included 7,497 cells grouped into 32 clusters. The total bone marrow yielded hematopoietic populations. Those populations’ depletion resulted in primarily erythroid progenitors with low expression of Cd45 and 2% non-hematopoietic cells. These rare populations were captured by depleting cells expressing CD45 or an erythroid marker CD71. The identified non-hematopoietic populations included Schwann cells, smooth muscle cells, myofibroblasts, Pdgfra^+^ mesenchymal cells, and endothelial cells. The endothelial subset was split into arterial and sinusoidal cells. The mesenchymal subset comprised nine sub-populations, including chondrocytes, osteoblasts, three fibroblast clusters, Ng2^+^Nestin^+^ MSCs, and two populations similar to Cxcl12-abundant reticular (CAR) cells. The two CAR sub-populations differed primarily in their expression of adipocyte (*Adipoq*) and osteo-lineage (*Sp7*, *Bglap*) genes. RNA velocity analysis of the mesenchymal lineage cells assigned the Ng2^+^ MSCs atop the differentiation hierarchy.

Baccin et al. (2020) additionally developed a laser-capture microdissection protocol coupled with sequencing (LCM-seq) to overcome spatial transcriptomic approaches that have not been successful in the bone marrow due to dependence on high RNA quality or unfixed tissue material. The method yielded full-length transcriptomic data from bone marrow sections containing 200-300 cells in a layer. LCM-seq was applied to 76 micro-dissected regions from the diaphyseal bone marrow to localize the bone marrow populations to endosteal, sinusoidal, arteriolar, and non-vascular niches. The frequencies of scRNA-seq populations within the spatially resolved transcriptomic data were estimated with CIBERSORT to determine the bone marrow cell types’ localization to candidate niches. Osteoblasts and chondrocytes localized to the endosteal niches. Arterial endothelial cells and smooth muscle cells were found in the arteriolar niches. Sinusoidal cells localized to the sinusoidal niches but were also detected in the (sub)-endosteal niches. Adipo-CAR cells were associated with high sinusoidal areas, but the Osteo-CAR cells were found in arteriolar or non-vascular niches. Additionally, to predict these cell types’ spatial relationships exclusively based on scRNA-seq, the RNA-Magnet method was developed, taking advantage of known cellular adhesion receptors and cognate ligands. The inferred adhesiveness of the identified populations to distinct niches correlated with the localization as measured by spatial transcriptomics. RNA-Magnet was also applied to signaling mediators, such as cytokines and growth factors, expressed by all cells in the dataset. The analysis suggested that the HSPCs were more likely to receive signals from non-hematopoietic populations than the mature immune cells, indicating a shift from mesenchymal to immune signaling. Both Adipo-CAR and Osteo-CAR cells were the primary sources of signals recognized by progenitor populations.

Zhong et al. (2020) performed additional profiling of mesenchymal lineage cells in young, adult, and aging mice. The study used a Col2-Cre mouse model as a strategy to capture the most primitive mesenchymal lineage cells. 13,759 cells with a median of 2,686 detected genes were captured from young (1-1.5-month-old) animals. Clustering analysis identified 22 populations, of which 9 were mesenchymal, 11 hematopoietic, 1 endothelial, and 1 mural. The 7,585 mesenchymal linage cells contained osteoblast, osteocyte, adipocyte, and chondrocyte populations as well as four progenitor subsets. Early mesenchymal progenitors (EMPs) were determined as the most primitive cells in the dataset based on the expression of stemness markers *Sca1*, *Cd34*, and *Thy1*. Intermediate mesenchymal progenitors and late mesenchymal progenitors expressed progressively higher levels osteo-lineage genes such as *Sp7* and *Col1a1*. Differentiation trajectory inference with Monocle placed EMPs at the terminal end of one of the three branches, with terminally differentiated osteocytes and adipocytes at the other ends. Lineage committed progenitors were placed at the point of bifurcation. Pseudotemporal reconstruction was additionally performed with Slingshot, which builds minimum spanning trees from clusters as opposed to single cells with Monocle. UMAP cell embeddings were used for trajectory inference, resulting in an analogous branching structure.

## Integrated Overview of the Bone Marrow Niche Datasets

**FIGURE 1:**
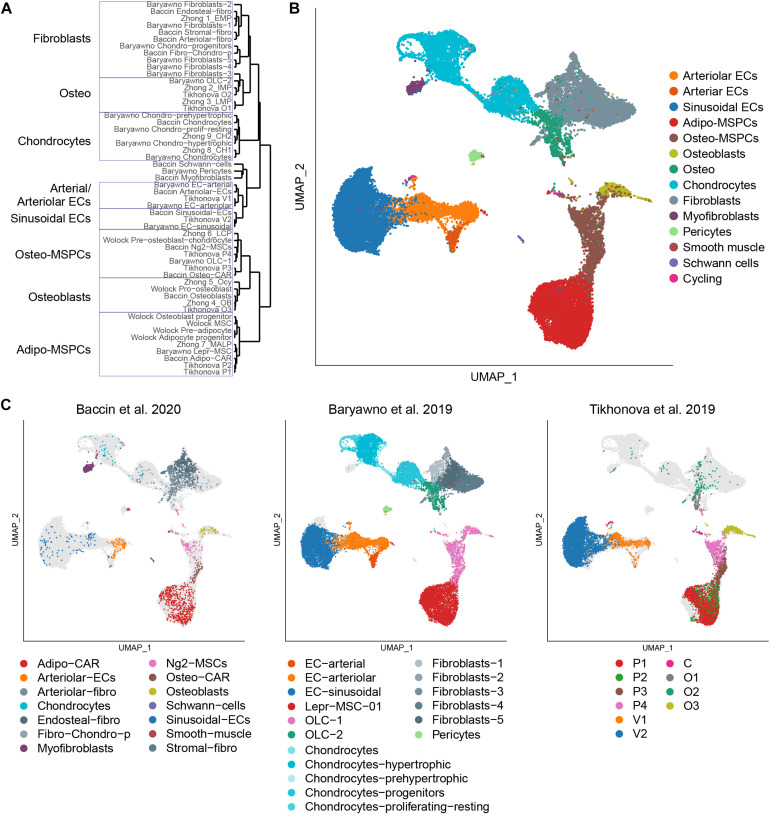
Integrated analysis of the bone marrow niche datasets. **(A)** Hierarchical clustering of the populations profiled in the discussed studies based on the scaled expression correlation distances of cluster marker genes. **(B)** UMAP visualization of all 32,743 cells from the integrated analysis, color-coded based on the harmonized population labels. **(C)** UMAP visualization of each of the datasets, color-coded based on the original population labels. Cells from the non-represented datasets are shown in light gray.

The single-cell studies have been able to reveal extensive heterogeneity in the populations thought to be homogeneous. One of the challenges associated with the rapid flow of this new information is an understanding of how the populations described in these studies are related. Furthermore, inconsistent nomenclature further complicates one’s ability to compare and contrast the data. To address this gap, we reanalyzed the five discussed scRNA-seq datasets combined (Table 1). We calculated the cluster markers for the populations identified within each dataset and performed hierarchical clustering based on the scaled expression correlation distances of those genes (Figure 1A). To better assess the heterogeneity of the described populations and their transcriptional relationships, we additionally performed a joint analysis of 32,743 cells from the three datasets encompassing the most diverse subsets. Using the Seurat anchor-based data integration pipeline (Stuart et al., 2019), we were able to overlay them and visualize the populations using a consistent representation (Figure 1B). Despite distinct sample preparation strategies (enzyme cocktails, digestion time, sorting and sorting strategies), assayed fraction (bone marrow or a combination of bone marrow and bone), and computational analysis approaches (summarized in Table 2), the findings are largely in agreement among the experiments carried out by independent groups. Expectantly, the relative abundances of various subpopulations fluctuated across the datasets (Figure 1C). For example, Baryawno dataset contained a large fraction of chondrocytes and fibroblasts, whereas Baccin subset uniquely captured myofibroblasts and Schwann cells.

**TABLE 2:** Computational approaches utilized by scRNA-seq studies profiling the bone marrow.

Operation	Tool/Algorithm	Description
Normalization, batch correction, clustering	Seurat (Butler et al., 2018; Stuart et al., 2019)	An R package for quality filtering, normalization, dimensionality reduction, and visualization of scRNA-seq data. It additionally includes a method for integrated analysis of multiple datasets by identifying pairwise correspondences between single cells across those datasets.
Visualization	t-SNE (van der Maaten and Hinton, 2008)	Non-linear dimensionality reduction technique based on Student-t distribution for converting data in a high-dimensional space to a low-dimensional one while avoiding overcrowding.
	SPRING (Weinreb et al., 2018a)	Force-directed graph layout for visualizing continuous topologies that generates a graph of nodes representing cells connected to their nearest neighbors in high-dimensional gene expression space.
	UMAP (Becht et al., 2018; McInnes et al., 2018)	Approximates a manifold and a constructs its fuzzy topological representation with the goal of preserving more of the global structure.
Pseudotime or trajectory inference	DPT/Destiny (Haghverdi et al., 2015; Angerer et al., 2016)	Uses diffusion maps to identify the low-dimensional structure, then identifies a pseudotime metric based on transition probabilities of differentiating toward various cell fates.
	Monocle (Trapnell et al., 2014; Qiu et al., 2017)	Constructs a minimum-spanning tree (MST) through the dimension-reduced space created by independent component analysis. Cells are ordered along the longest path through the MST. Monocle was later modified to use DDRTree for dimensionality reduction and ordering.
	PAGA (Wolf et al., 2019)	A partition-based graph abstraction tool that provides a coarse-grained representation by placing edges between cluster nodes with similar cells. Unlike many trajectory inference methods, it is able to account for disconnected topologies.
	PBA (Tusi et al., 2018; Weinreb et al., 2018b)	Uses nearest neighbor graph cell densities to predict fate probabilities and the direction of differentiation.
	Slingshot (Street et al., 2018)	Uses a cluster-based MST to identify the lineages and where they branch, then uses simultaneous principal curves to fit a smooth representations of each lineage.
	STEMNET (Velten et al., 2017)	Uses hierarchical clustering to define the most differentiated cell populations and then uses those populations as a training set for classifying priming in the less mature populations.
Velocity	Velocyto (La Manno et al., 2018)	Predicts the future state of single cells based on the relative abundance of unspliced precursor and spliced mature mRNA.
Cell-cell interactions	RNA-Magnet (Baccin et al., 2020)	Estimates the likelihood of physical interactions between single cells based on the expression of cell-surface receptors and their binding partners.

**FIGURE 2:**
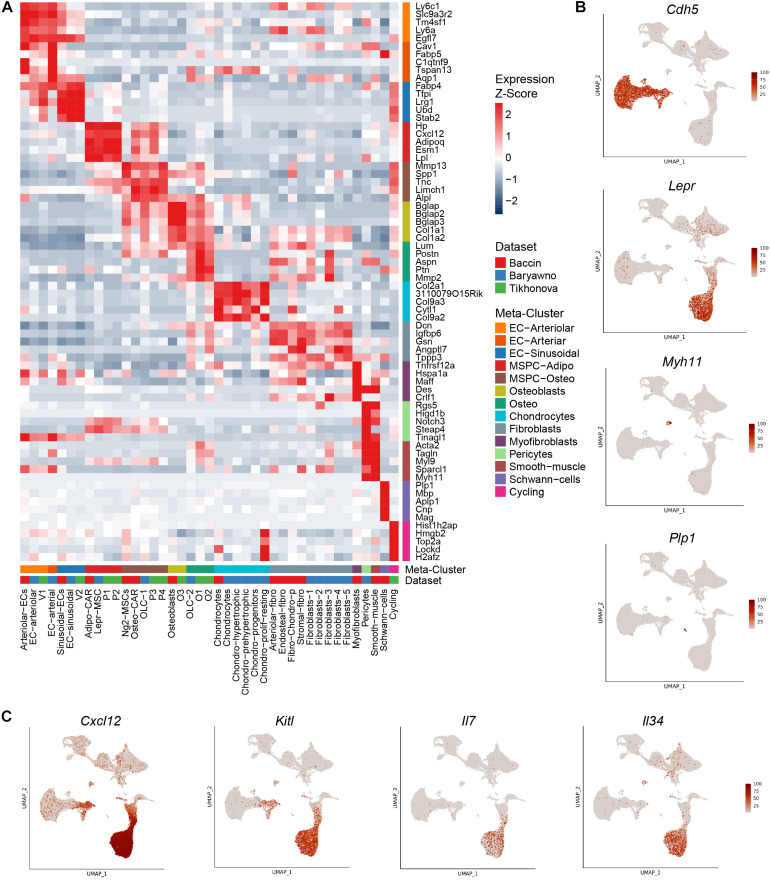
Expression patterns of marker genes and pro-hematopoietic factors. **(A)** Relative expression levels of the markers of the combined clusters within the populations defined in the original studies. **(B)** Expression levels of population marker genes *Cdh5* (ECs), *Lepr* (perivascular cells), *Myh11* (pericytes/smooth muscle), *Plp1* (Schwann cells) overlaid on the UMAP. **(C)** Expression levels of pro-hematopoietic factors *Cxcl12*, *Kitl*, *Il7*, and *Il34* overlaid on the UMAP.

Based on the expression patterns of cluster markers and visualization of integrated datasets, we harmonized the various sub-populations identified in the original studies using a consistent nomenclature (Figure 1B). Bone marrow vascular ECs were represented by sinusoidal (blue) and arteriolar (orange). By sampling a higher number of cells, Baryawno et al. identified an additional smaller sub-population of cells from endosteal and bone marrow arteries (dark orange). The mesenchymal stem cells give rise to a diverse range of cells such as adipocytes, osteoblasts, and chondrocytes. Previous studies took advantage of either *Cxcl12* or *Lepr* to isolate perivascular cells, but the extent of the overlap of the two populations was not clear. The scRNA-seq work revealed a broad transcriptional heterogeneity within these subsets. Bone marrow mesenchymal progenitors split into adipo-MSPCs (red) and osteo-MS (brown). Adipogenesis-primed mesenchymal cells were characterized by high expression of both *Lepr* and *Cxcl12*. These key markers were gradually decreased as the cells progressed toward osteogenesis-primed subpopulations. Osteoblasts (olive), chondrocytes (cyan) and a variety of fibroblast populations (gray) were represented across different dataset. Single cell studies are limited by the efficiency of digestion protocols and rare populations may not be sufficiently sampled. A more granular deconvolution of smaller bone subsets will require focused functional studies. To resolve transcriptional relationships between distinctly labeled clusters, the readers can refer to Figure 1A. To confirm that the gene expression of the integrated dataset corresponded to the populations identified in the individual studies, we assessed most differentially expressed marker genes (Figures 2A,B) and mapped expression patters of key pro-hematopoietic factors in the bone (Figure 2C). Indeed, we found the expression of *Cxcl12* and *Kitl* to be enriched in adipo-MSCPs progenitors and arteriolar ECs, while *Il7* and *Il34* were expressed primarily in adipo-MSPCs (Figure 2C). Collectively, our brief survey highlights a concordance between five different bone marrow niche single-cell datasets and resolves inconsistent nomenclature offered by distinct scientific groups.

## Zooming Into the Vascular Niches

Vascular ECs comprise a highly organized network throughout the body, facilitating the distribution of oxygen and nutrients. In addition to their delivery function, ECs enable immune cell trafficking allowing for cell migration and immune surveillance. Vascular ECs display tissue-specific morphology and have been shown in some cases to carry organ-specific functions. Blood-brain barrier and kidney glomeruli vasculature have been areas of active research, but the specifics of vascular cells in other tissues are less understood. High-throughput transcriptional profiling studies compared molecular architecture of ECs across various organs, revealing a marked transcriptional heterogeneity (Chi et al., 2003; Nolan et al., 2013). In addition to variability driven by tissues, each organ contains several vessel types, including arteries, veins, and capillaries. Recent single-cell RNA-seq analysis of > 32,000 ECs isolated from 11 different mouse tissues at the steady-state showed that vascular heterogeneity is largely dictated by the anatomical location of ECs rather than the vessel type (Kalucka et al., 2020), suggesting tissues-mediated molding of the vasculature and tissues specific functions.

**FIGURE 3:**
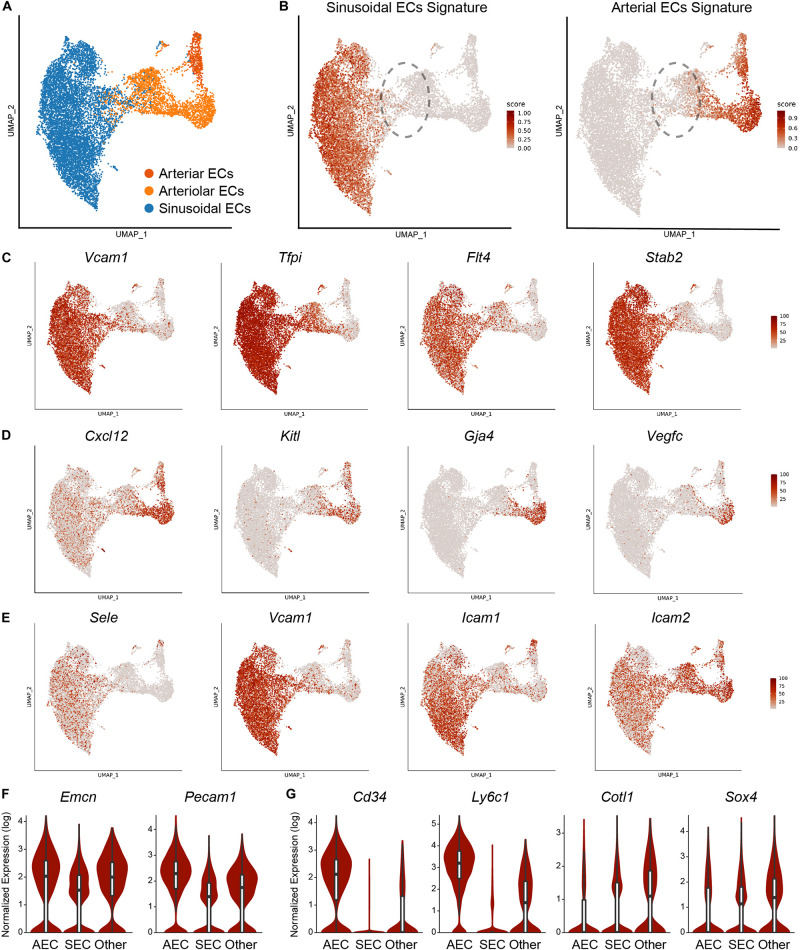
Analysis of the EC subset. **(A)** UMAP visualization of 10,578 ECs from the integrated analysis, color-coded based on the harmonized population labels. **(B)** Module scores for sinusoidal and arterial gene signatures overlaid on the UMAP. Dashed outline highlights the population not enriched for sinusoidal or arterial signatures. Expression of sinusoidal **(C)** and arteriolar **(D)** marker genes as well as adhesion molecules **(E)** overlaid on the UMAP representation. **(F)** Expression of transitional H vessel cell-surface markers within the populations defined by sinusoidal and arterial EC signatures. **(G)** Expression of marker genes within the populations defined by sinusoidal and arterial EC signatures.

In the bone marrow, ECs serve multiple roles, including HSC maintenance and leukocyte trafficking. Highly branched sinusoidal capillaries (SECs) carry venous blood and make up the vast majority of vascular cells. Arteriole vessels (AECs) carry arterial blood into the bone, have few branches, and constitute around ∼10% of the total bone marrow vasculature (Xu et al., 2018; Tikhonova et al., 2019). Here, we specifically focused on the vascular subset from the combined single-cell bone marrow niche datasets (Figure 3A). We calculated module scores for ECs based on the sinusoidal and arterial gene expression signatures obtained from the bulk tissue analysis (Xu et al., 2018) and found that 67.3% of cells were enriched for sinusoidal and 23.5% for the arterial signatures (Figure 3B). To further confirm the identity of the cells, we assessed the expression levels of population marker genes and found *Flt4*, *Tfpi*, *Stab2*, and *Vcam1* to be highly specific to sinusoids (Figure 3C) and *Cxl12, Kitl, Gja4, Vegfc* to arterioles (Figure 3D). Previous studies indicated that sinusoids and arterioles possess different permeability properties (Itkin et al., 2016). Notably, hematopoietic stem cell adhesion, rolling and trans-endothelial events were reported exclusively in SECs (Itkin et al., 2016). Indeed, sinusoids are enriched for expression of adhesion molecules such as E-selectin (*Sele*), Vcam-1 (*Vcam1*), ICAM-1 (*Icam1*), while arterioles expressed ICAM-2 (*Icam2*) (Figure 3E), consistent with the idea of differential trans-endothelial migration. It is not clear if all sinusoids can support trans-endothelial migration and it is tempting to speculate that there might be specialized sinusoid subtypes facilitating leukocyte trafficking.

Within the bone marrow, transitional zone vessels connect bone marrow sinusoids and arterioles. Based on the cell-surface marker expression and phenotypic characteristics, Kasumbe et al. identified two subtypes of SECs: CD31^low^EMCN^low^ L-vessels form highly branched capillary networks throughout the bone marrow and represent the majority of the SECs. CD31^high^EMCN^high^ H-vessels were found in the metaphysis, organized as vessel columns, and in transitional zones bridging arterioles with the sinusoids (Kusumbe et al., 2014). H-vessels are of a substantial clinical interest, as they were shown to regulate osteogenesis (Kusumbe et al., 2014, 2016; Ramasamy et al., 2014). We were not able to clearly identify transitional vessels with our own transcriptional dataset (Tikhonova et al., 2019). However, exploring the combined analysis based on the three studies allowed us to pinpoint a subset of ECs that were not enriched for either arterial or sinusoidal signatures. Further examination revealed that these transition cells expressed higher levels of *Emcn* and *Cd31* compared to sinusoids, suggesting that they might represent transitional H vessels (Figure 3F). We found this population to be enriched for arterial-associated genes Cd34 and Ly6c1 but also express unique markers such as *Cotl1* and *Sox4* (Figure 3G). Collectively, this integrated analysis allows for deeper exploration of BM EC heterogeneity. Although transitional EC population was not clearly revealed by each individual analysis, combining the datasets allowed for their further exploration, underscoring the utility of publicly available data to the community.

A recent study reports even further specification among bone marrow SECs. Chen and Liu et al. demonstrated that Apelin^+^ (*Apln*) bone marrow ECs control vascular regeneration following irradiation-induced vascular damage. These specialized SECs make up approximately 0.03% of the total bone marrow and are located in the metaphysis, endosteum, and the transition area between the metaphysis and diaphysis. Bulk RNA-seq of Apln^+^ cells revealed an enrichment of pathways related to angiogenesis and vascular endothelial growth factor (VEGF) signaling. Importantly, irradiation triggers the expansion of Apln^+^ ECs and vascular regeneration. Spatial organization of hematopoietic differentiation is another open question. Elegant work by Zhang et al. revealed that following acute systemic infection, monocyte–dendritic cell progenitors localize to a subset of blood vessels expressing colony-stimulating factor 1 (*Csf1*), known to be a key regulator of myelopoiesis. Indeed, EC-specific deletion of Csf1, results in a loss of non-classical monocytes and dendritic cells during steady-state and following infection (Zhang et al., 2021). It remains to be elucidated if specialized ECs also support progenitors of lymphoid and erythroid lineages. Further mining of single-cell bone marrow niche datasets, single-cell sequencing of ECs following stress scenarios, combined with receptor-ligand interaction analysis (Browaeys et al., 2019; Cabello-Aguilar et al., 2020; Efremova et al., 2020) and clever functional studies will reveal further specialization of vascular ECs.

The precise identity of the vascular cells that support HSCs continues to be debated. Combining computational modeling with whole-mount confocal immunofluorescence imaging techniques, Kunisaki et al. found quiescent HSCs to be closely associated with AECs (Kunisaki et al., 2013). Optically clearing studies of the bone marrow, that allowed to perform deep confocal imaging, demonstrate that independently of their cycling status, approximately 80% of HSCs are closely associated with SECs, with another 10% of HSCs being adjacent to AECs, and 10% being adjacent to transition zone vessels (Acar et al., 2015). In contrast, Kunisaki et al., using whole-mount confocal immunofluorescence imaging, showed that quiescent HSCs associate specifically with small arterioles that are preferentially found in endosteal bone marrow. Furthermore, Itkin et al. demonstrated that arterioles maintain hematopoietic stem cells in a low reactive species oxygen state (Itkin et al., 2016), perpetuating the debate regarding the precise identity of vascular cells that support quiescent HSCs. Just as novel technologies have been driving forward our understanding of hematopoietic differentiation, further advances combining multi-modal single-cell approaches, such as pairing gene expression data with spatial tissue context, and *in vivo* imaging will shape our understanding of hematopoietic cell interactions with their niches and resolve standing questions.

## Conclusion

In a span of less than two years, our understanding of the hematopoietic differentiation and bone marrow microenvironment has made a major leap forward. Despite these advances, key questions remain unanswered (Tikhonova et al., 2020). For example, cellular identity of the bone marrow mesenchymal stem cells remains to be elucidated. Despite highly comparable data, pseudo-temporal ordering revealed conflicting differential MSPC hierarchies and led to distinct conclusions. We believe that scRNA-seq analyses are able to guide our understanding of MSPC biology and their exploration can inspire creativity in our scientific inquiry. However, they cannot replace functional experiments required to answer complex biological questions.

Single-cell profiling of hematopoietic hierarchy highlighted a fluidic nature of differentiation. Although we understand some of the intrinsic signals implementing these decisions, we do not yet grasp which extrinsic factors mediate hematopoietic differentiation. We speculate that specific temporospatial combinations of bone marrow niche signals guide hematopoietic progenitors toward gradual commitment to a lineage choice. Novel computational approaches and genetic strategies are required to connect these dots.

## Data Availability Statement

An interactive browser of the integrated analysis presented in this study is available at the Broad Institute Single Cell Portal (https://singlecell.broadinstitute.org/single_cell/study/SCP1248). The data from the integrated analysis is available through the Open Science Framework (https://osf.io/ne9vj).

## Author Contributions

All authors listed have made a substantial, direct and intellectual contribution to the work, and approved it for publication.

## Conflict of Interest

The authors declare that the research was conducted in the absence of any commercial or financial relationships that could be construed as a potential conflict of interest.

## References

[B1] Acar M. Kocherlakota K. S. Murphy M. M. Peyer J. G. Oguro H. Inra C. N. (2015). Deep imaging of bone marrow shows non-dividing stem cells are mainly perisinusoidal. *Nature* 526 126–130. 10.1038/nature15250 26416744 PMC4850557

[B2] Angerer P. Haghverdi L. Büttner M. Theis F. J. Marr C. Buettner F. (2016). destiny: diffusion maps for large-scale single-cell data in R. *Bioinformatics* 32 1241–1243. 10.1093/bioinformatics/btv715 26668002

[B3] Baccin C. Al-Sabah J. Velten L. Helbling P. M. Grünschläger F. Hernández-Malmierca P. (2020). Combined single-cell and spatial transcriptomics reveal the molecular, cellular and spatial bone marrow niche organization. *Nat. Cell Biol.* 22 38–48. 10.1038/s41556-019-0439-6 31871321 PMC7610809

[B4] Baryawno N. Przybylski D. Kowalczyk M. S. Kfoury Y. Severe N. Gustafsson K. (2019). A cellular taxonomy of the bone marrow stroma in homeostasis and leukemia. *Cell* 177 1915–1932.e16. 10.1016/j.cell.2019.04.040 31130381 PMC6570562

[B5] Becht E. McInnes L. Healy J. Dutertre C.-A. A. Kwok I. W. H. H. Ng L. G. (2018). Dimensionality reduction for visualizing single-cell data using UMAP. *Nat. Biotechnol.* 37 38–47. 10.1038/nbt.4314 30531897

[B6] Browaeys R. Saelens W. Saeys Y. (2019). NicheNet: modeling intercellular communication by linking ligands to target genes. *Nat. Methods* 17 159–162. 10.1038/s41592-019-0667-5 31819264

[B7] Butler A. Hoffman P. Smibert P. Papalexi E. Satija R. (2018). Integrating single-cell transcriptomic data across different conditions, technologies, and species. *Nat. Biotechnol.* 36 411–420. 10.1038/nbt.4096 29608179 PMC6700744

[B8] Cabello-Aguilar S. Alame M. Kon-Sun-Tack F. Fau C. Lacroix M. Colinge J. (2020). SingleCellSignalR: inference of intercellular networks from single-cell transcriptomics. *Nucleic Acids Res.* 48:e55. 10.1093/nar/gkaa183 32196115 PMC7261168

[B9] Cabezas-Wallscheid N. Klimmeck D. Hansson J. Lipka D. B. Reyes A. Wang Q. (2014). Identification of regulatory networks in HSCs and their immediate progeny via integrated proteome, transcriptome, and DNA methylome analysis. *Cell Stem Cell* 15 507–522. 10.1016/j.stem.2014.07.005 25158935

[B10] Chi J. T. Chang H. Y. Haraldsen G. Jahnsen F. L. Troyanskaya O. G. Chang D. S. (2003). Endothelial cell diversity revealed by global expression profiling. *Proc. Natl. Acad. Sci. U.S.A.* 100 10623–10628. 10.1073/pnas.1434429100 12963823 PMC196854

[B11] Ding L. Morrison S. J. (2013). Haematopoietic stem cells and early lymphoid progenitors occupy distinct bone marrow niches. *Nature* 495 231–235. 10.1038/nature11885 23434755 PMC3600153

[B12] Ding L. Saunders T. L. Enikolopov G. Morrison S. J. (2012). Endothelial and perivascular cells maintain haematopoietic stem cells. *Nature* 481 457–462. 10.1038/nature10783 22281595 PMC3270376

[B13] Efremova M. Vento-Tormo M. Teichmann S. A. Vento-Tormo R. (2020). CellPhoneDB: inferring cell–cell communication from combined expression of multi-subunit ligand–receptor complexes. *Nat. Protoc.* 15 1484–1506. 10.1038/s41596-020-0292-x 32103204

[B14] Ghazanfari R. Li H. Zacharaki D. Lim H. C. Scheding S. (2016). Human non-hematopoietic CD271 pos/CD140a low/neg bone marrow stroma cells fulfill stringent stem cell criteria in serial transplantations. *Stem Cells Dev.* 25 1652–1658. 10.1089/scd.2016.0169 27527928 PMC5098131

[B15] Gomariz A. Helbling P. M. Isringhausen S. Suessbier U. Becker A. Boss A. (2018). Quantitative spatial analysis of haematopoiesis-regulating stromal cells in the bone marrow microenvironment by 3D microscopy. *Nat. Commun.* 9:2532. 10.1038/s41467-018-04770-z 29955044 PMC6023894

[B16] Haghverdi L. Buettner F. Theis F. J. (2015). Diffusion maps for high-dimensional single-cell analysis of differentiation data. *Bioinformatics* 31 2989–2998. 10.1093/bioinformatics/btv325 26002886

[B17] Itkin T. Gur-Cohen S. Spencer J. A. Schajnovitz A. Ramasamy S. K. Kusumbe A. P. (2016). Distinct bone marrow blood vessels differentially regulate haematopoiesis. *Nature* 532 323–328. 10.1038/nature17624 27074509 PMC6450701

[B18] Kalucka J. de Rooij L. P. M. H. Goveia J. Rohlenova K. Dumas S. J. Meta E. (2020). Single-cell transcriptome atlas of murine endothelial cells. *Cell* 180 764–779.e20. 10.1016/j.cell.2020.01.015 32059779

[B19] Kiel M. J. Yilmaz ÖH. Iwashita T. Yilmaz O. H. Terhorst C. Morrison S. J. (2005). SLAM family receptors distinguish hematopoietic stem and progenitor cells and reveal endothelial niches for stem cells. *Cell* 121 1109–1121. 10.1016/j.cell.2005.05.026 15989959

[B20] Kowalczyk M. S. Tirosh I. Heckl D. Rao T. N. Dixit A. Haas B. J. (2015). Single-cell RNA-seq reveals changes in cell cycle and differentiation programs upon aging of hematopoietic stem cells. *Genome Res.* 25 1860–1872. 10.1101/gr.192237.115 26430063 PMC4665007

[B21] Kunisaki Y. Bruns I. Scheiermann C. Ahmed J. Pinho S. Zhang D. (2013). Arteriolar niches maintain haematopoietic stem cell quiescence. *Nature* 502 637–643. 10.1038/nature12612 24107994 PMC3821873

[B22] Kusumbe A. P. Ramasamy S. K. Adams R. H. (2014). Coupling of angiogenesis and osteogenesis by a specific vessel subtype in bone. *Nature* 507 323–328. 10.1038/nature13145 24646994 PMC4943525

[B23] Kusumbe A. P. Ramasamy S. K. Itkin T. Mäe M. A. Langen U. H. Betsholtz C. (2016). Age-dependent modulation of vascular niches for haematopoietic stem cells. *Nature* 532 380–384. 10.1038/nature17638 27074508 PMC5035541

[B24] La Manno G. Soldatov R. Zeisel A. Braun E. Hochgerner H. Petukhov V. (2018). RNA velocity of single cells. *Nature* 560 494–498. 10.1038/s41586-018-0414-6 30089906 PMC6130801

[B25] Laurenti E. Göttgens B. (2018). From haematopoietic stem cells to complex differentiation landscapes. *Nature* 553 418–426. 10.1038/nature25022 29364285 PMC6555401

[B26] Liggett L. A. Sankaran V. G. (2020). Unraveling hematopoiesis through the lens of genomics. *Cell* 182 1384–1400. 10.1016/j.cell.2020.08.030 32946781 PMC7508400

[B27] McInnes L. Healy J. Melville J. (2018). UMAP: uniform manifold approximation and projection for dimension reduction. *arXiv* [Preprint]. Available online at: http://arxiv.org/abs/1802.03426 (accessed February 10, 2021).

[B28] Nestorowa S. Hamey F. K. Pijuan Sala B. Diamanti E. Shepherd M. Laurenti E. (2016). A single-cell resolution map of mouse hematopoietic stem and progenitor cell differentiation. *Blood* 128 e20–e31. 10.1182/blood-2016-05-716480 27365425 PMC5305050

[B29] Nolan D. J. Ginsberg M. Israely E. Palikuqi B. Poulos M. G. James D. (2013). Molecular signatures of tissue-specific microvascular endothelial cell heterogeneity in organ maintenance and regeneration. *Dev. Cell* 26 204–219. 10.1016/j.devcel.2013.06.017 23871589 PMC3873200

[B30] Paul F. Arkin Y. Giladi A. Jaitin D. A. Kenigsberg E. Keren-Shaul H. (2015). Transcriptional heterogeneity and lineage commitment in myeloid progenitors. *Cell* 163 1663–1677. 10.1016/j.cell.2015.11.013 26627738

[B31] Pellin D. Loperfido M. Baricordi C. Wolock S. L. Montepeloso A. Weinberg O. K. (2019). A comprehensive single cell transcriptional landscape of human hematopoietic progenitors. *Nat. Commun.* 10:2395. 10.1038/s41467-019-10291-0 31160568 PMC6546699

[B32] Pietras E. M. Reynaud D. Kang Y.-A. Carlin D. Calero-Nieto F. J. Leavitt A. D. (2015). Functionally distinct subsets of lineage-biased multipotent progenitors control blood production in normal and regenerative conditions. *Cell Stem Cell* 17 35–46. 10.1016/j.stem.2015.05.003 26095048 PMC4542150

[B33] Pinho S. Frenette P. S. (2019). Haematopoietic stem cell activity and interactions with the niche. *Nat. Rev. Mol. Cell Biol.* 20 303–320. 10.1038/s41580-019-0103-9 30745579 PMC6483843

[B34] Poulos M. G. Guo P. Kofler N. M. Pinho S. Gutkin M. C. Tikhonova A. (2013). Endothelial jagged-1 is necessary for homeostatic and regenerative hematopoiesis. *Cell Rep.* 4 1022–1034. 10.1016/j.celrep.2013.07.048 24012753 PMC3805263

[B35] Qiu X. Hill A. Packer J. Lin D. Ma Y.-A. Trapnell C. (2017). Single-cell mRNA quantification and differential analysis with Census. *Nat. Methods* 14 309–315. 10.1038/nmeth.4150 28114287 PMC5330805

[B36] Ramasamy S. K. Kusumbe A. P. Wang L. Adams R. H. (2014). Endothelial Notch activity promotes angiogenesis and osteogenesis in bone. *Nature* 507 376–380. 10.1038/nature13146 24647000 PMC4943529

[B37] Reya T. Morrison S. J. Clarke M. F. Weissman I. L. (2001). Stem cells, cancer, and cancer stem cells. *Nature* 414 105–111. 10.1038/35102167 11689955

[B38] Street K. Risso D. Fletcher R. B. Das D. Ngai J. Yosef N. (2018). Slingshot: cell lineage and pseudotime inference for single-cell transcriptomics. *BMC Genomics* 19:477. 10.1186/s12864-018-4772-0 29914354 PMC6007078

[B39] Stuart T. Butler A. Hoffman P. Hafemeister C. Papalexi E. Mauck W. M. (2019). Comprehensive integration of single-cell data. *Cell* 177 1888–1902.e21. 10.1016/j.cell.2019.05.031 31178118 PMC6687398

[B40] Sugiyama T. Kohara H. Noda M. Nagasawa T. (2006). Maintenance of the hematopoietic stem cell pool by CXCL12-CXCR4 chemokine signaling in bone marrow stromal cell niches. *Immunity* 25 977–988. 10.1016/j.immuni.2006.10.016 17174120

[B41] Tikhonova A. N. Dolgalev I. Hu H. Sivaraj K. K. Hoxha E. Cuesta-Domínguez Á (2019). The bone marrow microenvironment at single-cell resolution. *Nature* 569 222–228. 10.1038/s41586-019-1104-8 30971824 PMC6607432

[B42] Tikhonova A. N. Lasry A. Austin R. Aifantis I. (2020). Cell-by-cell deconstruction of stem cell niches. *Cell Stem Cell* 27 19–34. 10.1016/j.stem.2020.06.013 32619515 PMC8176558

[B43] Trapnell C. Cacchiarelli D. Grimsby J. Pokharel P. Li S. Morse M. (2014). The dynamics and regulators of cell fate decisions are revealed by pseudotemporal ordering of single cells. *Nat. Biotechnol.* 32 381–386. 10.1038/nbt.2859 24658644 PMC4122333

[B44] Tusi B. K. Wolock S. L. Weinreb C. Hwang Y. Hidalgo D. Zilionis R. (2018). Population snapshots predict early haematopoietic and erythroid hierarchies. *Nature* 555 54–60. 10.1038/nature25741 29466336 PMC5899604

[B45] van der Maaten L. Hinton G. (2008). Visualizing data using t-SNE. *J. Mach. Learn. Res.* 9 2579–2605.

[B46] Velten L. Haas S. F. Raffel S. Blaszkiewicz S. Islam S. Hennig B. P. (2017). Human haematopoietic stem cell lineage commitment is a continuous process. *Nat. Cell Biol.* 19 271–281. 10.1038/ncb3493 28319093 PMC5496982

[B47] Watcham S. Kucinski I. Gottgens B. (2019). New insights into hematopoietic differentiation landscapes from single-cell RNA sequencing. *Blood* 133 1415–1426. 10.1182/blood-2018-08-835355 30728144 PMC6440294

[B48] Weinreb C. Wolock S. Klein A. M. (2018a). SPRING: a kinetic interface for visualizing high dimensional single-cell expression data. *Bioinformatics* 34 1246–1248. 10.1093/bioinformatics/btx792 29228172 PMC6030950

[B49] Weinreb C. Wolock S. Tusi B. K. Socolovsky M. Klein A. M. (2018b). Fundamental limits on dynamic inference from single-cell snapshots. *Proc. Natl. Acad. Sci. U.S.A.* 115 E2467–E2476. 10.1073/pnas.1714723115 29463712 PMC5878004

[B50] Weissman I. L. (2000). Stem cells: units of development, units of regeneration, and units in evolution. *Cell* 100 157–168. 10.1016/S0092-8674(00)81692-X 10647940

[B51] Wolf F. A. Hamey F. K. Plass M. Solana J. Dahlin J. S. Göttgens B. (2019). PAGA: graph abstraction reconciles clustering with trajectory inference through a topology preserving map of single cells. *Genome Biol.* 20:59. 10.1186/s13059-019-1663-x 30890159 PMC6425583

[B52] Wolock S. L. Krishnan I. Tenen D. E. Matkins V. Camacho V. Patel S. (2019). Mapping distinct bone marrow niche populations and their differentiation paths. *Cell Rep.* 28 302–311.e5. 10.1016/j.celrep.2019.06.031 31291568 PMC6684313

[B53] Xu C. Gao X. Wei Q. Nakahara F. Zimmerman S. E. Mar J. (2018). Stem cell factor is selectively secreted by arterial endothelial cells in bone marrow. *Nat. Commun.* 9:2449. 10.1038/s41467-018-04726-3 29934585 PMC6015052

[B54] Zhang J. Wu Q. Johnson C. B. Pham G. Kinder J. M. Olsson A. (2021). In situ mapping identifies distinct vascular niches for myelopoiesis. *Nature.* 590, 457–462. 10.1038/s41586-021-03201-2 33568812 PMC8020897

[B55] Zheng S. Papalexi E. Butler A. Stephenson W. Satija R. (2018). Molecular transitions in early progenitors during human cord blood hematopoiesis. *Mol. Syst. Biol.* 14:e8041. 10.15252/msb.20178041 29545397 PMC5852373

[B56] Zhong L. Yao L. Tower R. J. Wei Y. Miao Z. Park J. (2020). Single cell transcriptomics identifies a unique adipose lineage cell population that regulates bone marrow environment. *Elife* 9:e54695. 10.7554/eLife.54695 32286228 PMC7220380

